# Characterization and Immunogenicity of HIV Envelope gp140 Zera^®^ Tagged Antigens

**DOI:** 10.3389/fbioe.2020.00321

**Published:** 2020-04-09

**Authors:** Phindile Ximba, Rosamund Chapman, Ann E. Meyers, Emmanuel Margolin, Michiel T. van Diepen, Anna-Lise Williamson, Edward P. Rybicki

**Affiliations:** ^1^Division of Medical Virology, Department of Pathology, Faculty of Health Sciences, University of Cape Town, Cape Town, South Africa; ^2^Biopharming Research Unit, Department of Molecular and Cell Biology, University of Cape Town, Cape Town, South Africa; ^3^Institute of Infectious Disease and Molecular Medicine, Faculty of Health Sciences, University of Cape Town, Cape Town, South Africa

**Keywords:** HIV-1, protein body, envelope protein, vaccine, Zera^®^, immunogenicity

## Abstract

HIV-1 envelope glycoprotein (Env) remains the most relevant target for the elicitation of functional antibodies to HIV by vaccination. However, soluble Env antigens often do not elicit the desired immune responses. Delivering subunit antigens on particulate nanoparticles is an established approach to improve their immunogenicity. In this study the sequence encoding Zera^®^, a proline-rich domain derived from the γ-zein storage protein, was fused to either the C- or N-terminus of the superinfecting HIV-1 CAP256 gp140 envelope: Zera^®^ generally induces the formation of protein bodies (PBs), which can significantly improve both the immunogenicity and yields of the partner protein. The expression of gp140-Zera^®^ and Zera^®^-gp140 (N- and C-terminal fusions respectively) in mammalian cells was confirmed by western blot analysis and immunostaining. However, isopycnic ultracentrifugation showed that neither gp140-Zera^®^ nor Zera^®^-gp140 accumulated in characteristic electron-dense PBs. gp140-Zera^®^ elicited higher binding antibody titers in rabbits to autologous gp140 and V1V2 scaffold than Zera^®^-gp140. Rabbit anti-gp140-Zera^®^ sera also had significantly higher Tier 1A neutralizing antibody titers than anti-Zera^®^-gp140 sera. Neither gp140-Zera^®^ nor Zera^®^-gp140-specific sera neutralized Tier 1B or autologous Tier 2 viruses. These results showed that HIV-1 gp140 tagged with Zera^®^ at either the N- or C-termini elicited high titers of gp140 and V1V2 binding antibodies, and low levels of Tier 1 neutralizing antibodies in rabbits.

## Introduction

An effective prophylactic vaccine remains an urgent priority to combat the HIV-1 pandemic. Theoretically, a preventative vaccine could work by inducing high titers of antibodies against the HIV-1 envelope glycoprotein (Env) with the capacity to neutralize the virus, and/or mediate effector functions to kill infected cells (Rerks-Ngarm et al., 2009; McCoy and Weiss, 2013; Burton and Hangartner, 2016; Alter and Barouch, 2018). In support of this, Pauthner et al. (2019) found macaques vaccinated with recombinant native-like Env trimers, that developed high titers of Tier 2 neutralizing antibodies, were protected against a matching SHIV challenge (Pauthner et al., 2019). A vaccine regimen consisting of a recombinant canarypox virus prime followed by an alum-adjuvanted HIV-1 subtype B/E bivalent gp120 boost conferred a modest 31% protection against HIV-1 acquisition in a Thai RV144 trial (Rerks-Ngarm et al., 2009). Vaccine-induced IgG antibodies to the gp120 V1V2 regions inversely correlated with the risk of HIV-1 acquisition (Haynes et al., 2012; Rolland and Gilbert, 2012). Additionally, non-neutralizing anti-Env antibodies (nNAbs) capable of mediating fragment crystallizable (Fc)-mediated antibody-dependent cellular cytotoxicity (ADCC) and weak Tier 1 neutralizing antibodies (NAbs) were secondary correlates of protection against HIV-1 acquisition (Bonsignori et al., 2012; Tomaras et al., 2013). There is an accumulating body of evidence that implicates Fc effector functions of both broadly neutralizing antibodies (bNAbs) and nNAbs in protection (Barouch et al., 2013; Bradley et al., 2017; Mayr et al., 2017; Alter and Barouch, 2018; Richardson et al., 2018). The protective efficacy conferred by vaccine-induced polyfunctional effector functions of nNAbs do not negate the need to design immunogens that elicit bNAb responses, but suggest that both nNAbs and bNAbs activities could produce synergistic effects in the development of protective responses against HIV-1 acquisition (Alter and Barouch, 2018; Lee and Kent, 2018).

Much research has been focused on the design of HIV-1 envelope immunogens that resemble native trimeric virion-associated proteins, that will elicit bNAbs. This includes the development of soluble precursor cleavage-dependent SOSIP trimers and cleavage-independent native flexibly linked (NFL) trimers (Sanders et al., 2013; Sharma et al., 2015). While these trimers are promising immunogens, soluble proteins are often poorly immunogenic and elicit rapidly waning immunity (Sliepen et al., 2015; Ingale et al., 2016; Bale et al., 2017; Brouwer et al., 2019; Sliepen et al., 2019). Multimerizing antigens as repeating arrays on the surface of virus-like particles or synthetic nanoparticles promotes efficient B cell activation and uptake by antigen-presenting cells for delivery to the lymph nodes, thus eliciting superior immune responses compared to soluble immunogens (Bachmann and Jennings, 2010). The presentation of HIV Env on nanoparticles is motivated by the success of the virus-like particle (VLP) vaccines that are on the market against human papillomaviruses and hepatitis E and B viruses (Zhao et al., 2013). Protein-based self-assembling nanoparticle platforms that have been explored for the presentation of Env-derived immunogens include lumazine synthase, dihydrolipoly acetyltransferase (E2p), a computationally designed hyperstable 60-mer (I3-01) and ferritin (Jardine et al., 2013; Sliepen et al., 2015; He et al., 2016; Georgiev et al., 2018; He et al., 2018). Fusion of the HIV-1 Clade A BG505 isolate-derived Env gp140 trimer to ferritin yielded nanoparticles displaying eight copies of the glycoprotein trimer, which elicited significantly higher autologous Tier 2 neutralizing antibodies than the corresponding soluble antigen (Sliepen et al., 2015). These results support the concept of using nanoparticle display platforms to develop HIV-1 Env vaccines with improved immunogenicity.

Genetically fusing antigens to protein body (PB)-producing fusion tags such as elastin-like polypeptides (derived from animal proteins), hydrophobin (fungal protein) and zein (plant protein) is another strategy to improve their immunogenicity, while also improving their accumulation and recovery (Conley et al., 2011). The use of a zein-derived tag for PB formation is especially promising, and it has been used as a genetic fusion to various antigens including HIV Nef (de Virgilio et al., 2008), a HPV type 16 E7 protein (Whitehead et al., 2014), an influenza virus A matrix 2 protein ectodomain (M2e) (Mbewana et al., 2015), the ectodomain of influenza A haemagglutinin (HA) subtype 5 (Hofbauer et al., 2016), bluetongue virus VP2 serotype-specific antigen (van Zyl et al., 2017), *Yersinia pestis* F1-V (Alvarez et al., 2010), xylanase (Llop-Tous et al., 2010) and various fluorescent proteins (Torrent et al., 2009b; Joseph et al., 2012; Hofbauer et al., 2014; Saberianfar et al., 2015). Zera^®^ (γ-zein ER-accumulating domain) is a 112 amino acid domain derived from the N-terminus of γ-zein which comprises the CGC motif downstream of the signal peptide, a central proline-rich domain containing a hexapeptide repeat (PPPVHL)_8_, as well as a C-terminal Pro-X domain with 4 cysteine residues (Geli et al., 1994). Zera^®^ induces the formation of electron-dense spherical protein body-like structures (1–2 μm) encapsulating large amounts of the protein of interest when fused to a heterologous protein. This has been reported for expression in a wide range of different hosts including plant, fungal, insect and mammalian cells (Llop et al., 2006; Torrent et al., 2009a, b; Conley et al., 2011; Whitehead et al., 2014; Mbewana et al., 2015; Hofbauer et al., 2016). The mechanisms by which γ-zeins drive self-assembly into PBs in the ER are not well understood as the sequence does not contain an obvious ER-retention signal. It is believed that the hydrophobic interactions between the amphipathic (PPPVHL)_8_ repeats and the formation of disulfide bonds between Zera^®^ molecules are characteristic features that allow for self-assembly into protein bodies (Kogan et al., 2001; Torrent et al., 2009b; Llop-Tous et al., 2010). The benefits of packaging the protein of interest in Zera^®^-induced PBs include the retention of protein in the ER (thus providing insulation against proteolysis in the cytoplasm), ease of purification as electron dense PBs allow for simple protein recovery using gradient centrifugation, and the adjuvanting effect of the particulate PBs (Torrent et al., 2009a; Schmidt, 2013; Whitehead et al., 2014). Encouragingly, the ectodomain of influenza HA fused to zein (H5-zein) formed protein bodies in tobacco leaves which were significantly more immunogenic in mice than the soluble H5 HA (Hofbauer et al., 2016). The adjuvant effect of H5-zein protein bodies was similar to the response elicited when soluble H5 was co-administered with a commercial adjuvant. Moreover, when H5-zein was co-administered with a commercial adjuvant, the H5-zein immune responses could not be enhanced, suggesting that the particulate nature of zein protein bodies was sufficient to mediate adjuvant effect.

It should be noted, however, that not all antigens of interest fused to zein accumulate in PBs (de Virgilio et al., 2008; Ceresoli et al., 2016), an indication that the properties of the protein of interest need careful consideration to increase the likelihood of benefiting from properties inherent to zein PBs.

In this study, we generated HIV-1 CAP256 gp140 with Zera^®^ fused to either the C-terminus (gp140-Zera^®^) or N-terminus (Zera^®^-gp140) and evaluated the formation of protein bodies in mammalian cells. The immunogenicity of these proteins was compared in rabbits in the absence of adjuvants to assess their ability to elicit Env and V1V2 binding antibodies.

## Materials and Methods

### CAP256 gp140-FL-IP, CAP256 SU V1V2 Scaffold, Antibodies, Plasmids, Cell Lines and Reagents

HIV-1 CAP256 gp140-FL-IP (hereafter referred to as gp140) protein was prepared as previously described (van Diepen et al., 2018). Goat anti-HIV-1 gp120 (Bio-Rad, 5000-0557), rabbit polyclonal antibody to calnexin (Abcam), mouse monoclonal anti-goat/sheep IgG-alkaline phosphatase (AP) GT34 (Sigma), donkey anti-goat IgG-Cy3 (Life Technologies), and donkey anti-rabbit IgG-Alexa 488 (Life Technologies) were used for western blots or immunofluorescence staining of fixed cells. HEK293T (ATCC^®^ USA, CRL-3216^TM^), HEK293 (ATCC^®^ USA, CRL-1573^TM^) and HeLa (ATCC^®^ USA, CCL-2^TM^) cells were cultured in Dulbecco’s modified Eagle medium (DMEM) (high glucose) plus L-glutamine (Lonza) supplemented with heat-inactivated 10% fetal calf serum and penicillin-streptomycin (Pen-Strep) (Gibco). All genes expressed in this study were cloned into the enhanced expression vector pTHpCapR backbone (Tanzer et al., 2011). CAP256 SU V1V2 scaffold protein was provided by Professor Penny Moore (Senior Medical Scientist, Centre for HIV and STIs, National Institute for Communicable Diseases, Johannesburg) and prepared as previously described (van Diepen et al., 2018).

### Generation of Mammalian Vectors That Express gp140-Zera^®^, Zera^®^-gp140, and Zera^®^-eGFP

To generate a plasmid expressing HIV-1 gp140 with a C-terminal Zera^®^ tag, the sequence coding for Zera^®^ (ZIP Solutions, Spain) appended with a flexible-linker (FL) on its 5′ end and the KDEL endoplasmic reticulum (ER) retention signal on its 3′ end, was human codon optimized, synthesized by GenScript and inserted into plasmid pMExT gp140-FL-IP (van Diepen et al., 2018), downstream of gp140, to generate pMExT gp140-Zera^®^. A plasmid expressing HIV gp140 with a N-terminal Zera^®^ tag (pMEx Zera^®^-gp140) was constructed by replacing the tissue plasminogen activator leader sequence of pMExT gp140-FL-IP with the Zera^®^ sequence. As a control, plasmid pMEx Zera^®^-eGFP, that expresses Zera^®^ fused to the 5′ end of the enhanced green fluorescent protein (eGFP) was constructed.

### Generation of Stable Cell Lines and Western Blotting

Plasmids for generating stable cell lines were constructed by inserting an IRES-Neomycin resistance cassette directly downstream of the Zera^®^ and gp140 sequences in pMExT gp140-Zera^®^ and pMEx Zera^®^-gp140 respectively. Stable cell lines were then generated by transfecting these plasmids into HEK293 cells and passaging at least 10 times in complete medium supplemented with 600 μg/ml geneticin (Gibco^TM^, Thermo Fisher Scientific). The cell media (M) and lysates (L) from transiently transfected cells or stable cell lines were analyzed on SDS-PAGE and transferred onto PVDF membranes (Bio-Rad, Hercules) for western blotting. The membranes were probed with the primary antibody [1:1000 goat anti-HIV-1 gp120 (Bio-Rad) or 1:4000 mouse anti-GFP (Sigma-Aldrich) or 1:5000 rabbit anti-Zera^®^ serum]. This was followed by incubation in appropriate secondary antibody conjugates [1:10 000 anti-goat/sheep-alkaline phosphatase (Sigma-Aldrich) or goat anti-mouse alkaline phosphatase (Sigma-Aldrich) or anti-rabbit alkaline phosphatase (Sigma-Aldrich), respectively]. The BCIP/NBT Phosphatase Substrate (KPL, Milford) was used for detection.

### Immunofluorescence Staining and Imaging

Immunofluorescent staining was conducted to evaluate the formation and localization of Zera^®^-induced protein bodies. Four-well chamber slides (AEC Amersham) were pre-coated with poly-L-Lysine (Sigma-Aldrich) and seeded with 30000 HeLa cells per well. Three days post transfection with pMExT gp140-Zera^®^, pMEx Zera^®^-gp140, pMExT gp140 or pMEx Zera^®^-eGFP, cells were fixed with 4% paraformaldehyde for 10 min, washed with 1x phosphate buffered saline (PBS), permeabilized and blocked with 2% BSA-PBS supplemented with 0.25% Triton X-100 for 1 h at room temperature. Cells were double-stained overnight at room temperature with goat anti-HIV-1 gp120 and rabbit polyclonal antibodies to calnexin (Abcam), diluted 1:500 and 1:200, respectively in 2% BSA-PBS. Following washes with PBS, cells were incubated in the dark for 1.5 h with the fluorophore-conjugated secondary antibodies (1:500 donkey anti-Goat-Cy3 and 1:500 donkey anti-rabbit-Alexa488/Cy3) diluted in 2% BSA-PBS. The cells were then washed with PBS and incubated for 10 min with 1:5000 Hoechst nuclei stain diluted in PBS. Following washes in PBS, slides were mounted with antifade-moviol mounting media. Slides were imaged with a confocal microscope (Carl Zeiss 880 LSM confocal with Fast Airyscan technology and the Elyra S1 super-resolution microscope).

### Preparation of Cell Lysates, Isopycnic Ultracentrifugation, Pelleting by Ultracentrifugation and Quantification of gp140

Transiently transfected HEK293T cells or stable HEK293 cell lines expressing gp140-Zera^®^, Zera^®^-gp140, Zera^®^-eGFP and gp140 were collected in the presence of Zera^®^ buffer PBP3 (100 mM Tris pH8, 50 mM KCl, 6 mM MgCl_2_, 10 mM EDTA, 0.4 M NaCl) supplemented with 1x cOmplete^TM^, EDTA-free Protease Inhibitor Cocktail (Sigma-Aldrich) and 10% sucrose. Cells were chilled on ice followed by homogenization through 15 up and down strokes of a Dounce glass tissue homogenizer (Kontes Glass Co., Vineland). Cell lysis was monitored by staining with Trypan Blue. Lysates were clarified by low-speed centrifugation (1000 × *g*, 5 min, 4°C) followed by filtration through 1 layer of sterile Miracloth (Merck) to remove floating cell debris. Clarified lysates were loaded on a 5–35% OptiPrep^TM^ continuous gradient prepared using a dual pump gradient maker (TRIS^TM^, ISCO, Lincoln, United Kingdom) followed by isopycnic ultracentrifugation (SW32Ti, 175000 × *g*, 16 h, 4°C). Gradients were manually fractionated by punching a hole in the bottom of the tube and slowly collecting 1ml fractions. Fractions were analyzed on a western blot probed with an antibody to Env. The Brix% of each fraction was measured using an ATAGO PAL-3 refractometer (0–93%, Brix) and converted to refractive indices and densities of OptiPrep^TM^. Band intensities of each fraction were plotted against the densities of OptiPrep^TM^ to determine the density distribution of gp140-Zera^®^ in comparison to Zera^®^-gp140. For immunogenicity studies, Zera^®^-tagged proteins from the clarified lysates were concentrated by pelleting through ultracentrifugation (SW28, 79000 × *g*, 2 h, 4°C). Protein pellets were resuspended in sterile PBS. The amount of gp140 in the pellets was estimated by densitometry analysis (Molecular Imager Gel Doc^TM^ XR + imaging system software, Bio-Rad) of samples run on a western blot. Gp140, purified by size exclusion chromatography and quantitated using the DC^TM^ Protein Assay (Bio-Rad) (van Diepen et al., 2019) was used as a standard.

### Immunization of Rabbits With gp140-Zera^®^ and Zera^®^-gp140 Proteins

Rabbit inoculations and blood sampling were performed at University of Stellenbosch Animal Research Facility in accordance with requirements, guidelines and approval of the University of Cape Town and University of Stellenbosch Animal Ethics Committees (AEC 015-51). Two groups of five female New Zealand White rabbits were used to compare the immunogenicity of the gp140-Zera^®^ and Zera^®^-gp140 proteins. Forty to 50 μg protein diluted in 1x PBS to a final volume of 500 μl, was administered into the quadricep muscle of the hind leg of each rabbit on day 0 and subsequently at weeks 4, 12, and 20 post initial immunization. Blood samples were collected into VACUETTE^®^ Z Serum Sep Clot Activator tubes (Greiner Bio-One) at weeks 0, 4, 8, 12, 14, 16, 20, and 22 post vaccination.

### Quantification of gp140 Binding Antibodies

An ELISA assay was used to quantify the gp140 antibody binding titers in rabbit sera as described previously (van Diepen et al., 2018). Briefly, 96-well Maxisorb^®^ microtiter ELISA plates (NunC) were coated with 10 ng/well of the soluble gp140 trimers (van Diepen et al., 2019). Duplicate wells were incubated with serum three-fold serially diluted from 1:10. Following secondary incubations with swine anti-rabbit IgG-HRPO conjugate (DakoCytomation) and detection with TMB ELISA substrate (Abcam^®^), the reaction was stopped after 10 min with 1N H_2_SO_4_. The absorbance signals at 450 nm were measured using a VersaMax ELISA Microplate Reader (Molecular Devices, Sunnyvale). Antibody end-point titers were defined as the last dilution to give a signal above the matching pre-bleed (1:10) ELISA signal. GraphPad Prism 5.0 software was used to present the end-point titres against bleeds time points (weeks). The V1V2-specific antibodies in sera collected at week 0 (pre-immune) and week 22 (2 weeks after the final protein inoculation) were evaluated as described above except that 500 ng/well of CAP256 SU V1V2 scaffold protein was used as a capture antigen.

### Neutralization Assays

A small panel of clade C Env-pseudotyped viruses competent for a single-cycle infection were used to evaluate the ability of anti-gp140 antibodies in serum sampled from weeks 0 and 22 to block the viral entry into susceptible TZM-bl cells. The degree of neutralization, measured in relative luminescence units (RLUs), was assayed by quantifying the reduction of Tat-regulated luciferase expression upon infection with Env-pseudotyped viruses (MW965.26, 6644 and CAP256SU). As a negative control, Env-pseudotyped virus containing Env of murine leukemia virus (MLV) encoded in the same backbone was included. The final neutralization titers were expressed as the reciprocal of the serum dilution resulting in a 50% reduction in relative luciferase units, or the ID_50_.

## Results

### Characterization of HIV-1 Subtype C CAP256 SU gp140 Fused to Zera^®^

**FIGURE 1:**
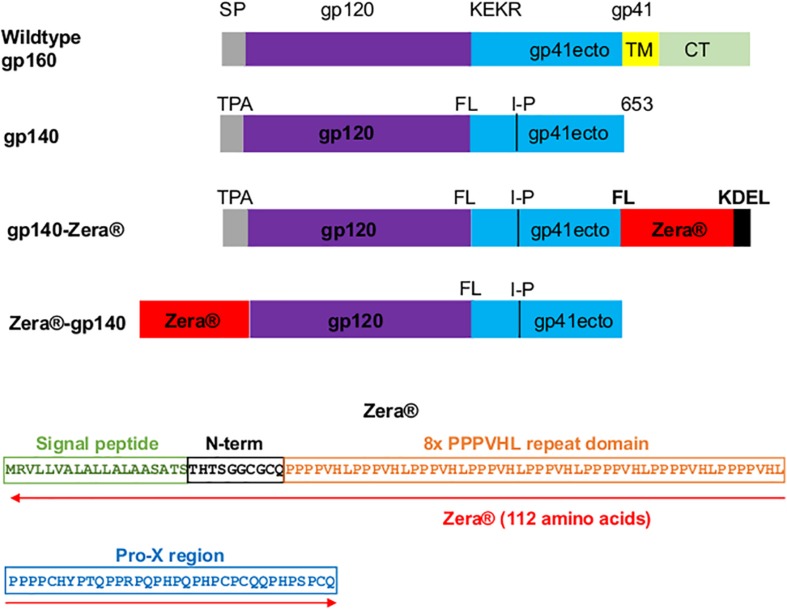
Design of Zera^®^-tagged proteins. Schematic representations of wildtype gp160, gp140, gp140-Zera^®^ and Zera^®^-gp140. The gp160 sequence was truncated at amino acid residue 653 to generate gp140, the native signal peptide sequence (SP) was replaced with the human tissue plasminogen activator leader sequence (TPA), the furin cleavage motif, KEKR, was replaced with a flexible linker (FL) and an I548P (I-P) mutation was introduced. The gp41 ectodomain (ecto), transmembrane (TM) and cytoplasmic domain (CT) are indicated. The amino acid sequence of Zera^®^ composed of the γ-Zein signal peptide, the non-proline N-terminal region with a CGC motif, the repeating domain containing eight units of the PPPVHL hexapeptide and the Pro-X C-terminal domain with four cysteine residues (the cysteine residues are underlined).

The CAP256 superinfecting viral envelope (CAP256 SU), modified as described previously by van Diepen et al. (2018), was used to generate plasmids encoding gp140-Zera^®^ and Zera^®^-gp140 (Figure 1). The pTHpCapR mammalian expression vector (Tanzer et al., 2011) was used as a plasmid backbone for both constructs. This plasmid contains a porcine circovirus-derived enhancer element upstream of the cytomegalovirus immediate early promoter which drives increased recombinant protein expression.

**FIGURE 2:**
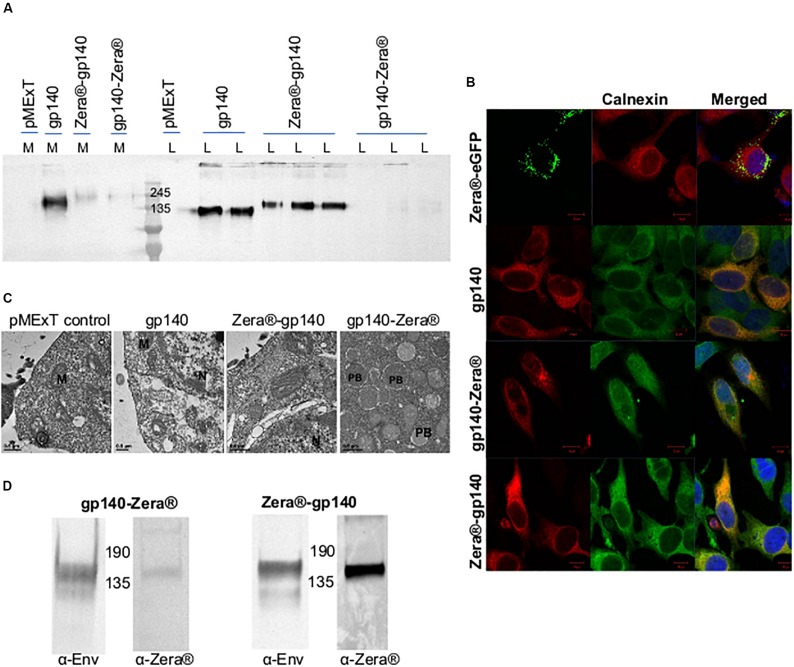
Characterization of Zera^®^-tagged proteins **(A)** Anti-Env western blot of the cell lysates (L) and media (M) from HEK293T cells transiently transfected with pMExT, pMExT gp140-FL-IP, pMExT gp140-Zera^®^ and pMEx Zera^®^-gp140. **(B)** Confocal micrographs of co-localization (merged) of Zera^®^-eGFP (green, eGFP fluorescence), gp140-Zera^®^ (red, Cy3) and Zera^®^-gp140 (red, Cy3) with calnexin (green, Alexa488) or red (Cy3) in HeLa cells. Cell nuclei were detected with a Hoechst stain (Blue). Scale bars represent 10 μM. **(C)** Electron micrographs of HEK293T cells transfected with pMExT, pMExT gp140-FL-IP, pMEx Zera^®^-gp140 and pMExT gp140-Zera^®^. M, mitochondrion; N, nucleus; and PB, protein body. **(D)** Anti-Env and anti-Zera^®^ western blot analysis of HEK293 stable cell lines expressing gp140-Zera^®^ and Zera^®^-gp140.

The expression of gp140, Zera^®^-gp140 and gp140-Zera^®^ in transiently transfected HEK293T cells was confirmed by western blot analysis of the cell media (M) and cell lysates (L) (Figure 2A). Transfection of HEK293T cells with pMExT gp140 and pMEx Zera^®^-gp140 resulted in the accumulation of similar levels of gp140 protein in the cell lysates (*n* > 3) whereas there was minimal accumulation of gp140 in the cell media and lysates from cells transfected with pMExT gp140-Zera^®^. The presence of Zera^®^ seemed to favor the accumulation of gp140 within the cells rather than secretion into the cell media.

The ability of the Zera^®^ tag to induce ER-derived gp140 protein bodies was evaluated by immunofluorescent staining and confocal microscopy (Figure 2B). HeLa cells were transiently transfected with pMExT gp140-Zera^®^ and pMEx Zera^®^-gp140, stained with antibodies to HIV-1 Env and calnexin (a chaperone located in the ER) and detected with Cy3 and Alexa-488-labeled secondary antibodies respectively. Cells transfected with pMEx Zera^®^-eGFP or pMExT gp140-FL-IP were included as positive and negative controls, respectively. Spherical protein body-like fluorescent structures were observed in cells expressing Zera^®^-eGFP and gp140-Zera^®^. It was noted that all the Zera^®^-eGFP appeared to be in protein bodies whereas gp140-Zera^®^ appeared to be a mixture of few, relatively small protein bodies as well as some soluble protein (diffused staining of the cell). Both Zera^®^-eGFP and gp140-Zera^®^ partially co-localized with the calnexin ER marker. Surprisingly, cells expressing Zera^®^-gp140 showed very similar staining to those expressing the untagged soluble, g140 protein and most of the gp140-Zera^®^. Both Zera^®^-gp140 and gp140 proteins also co-localized with the calnexin ER marker, an observation that reflected both intracellular and extracellular gp140 accumulation observed in western blot analysis shown in Figure 2A. A plasmid pMEx gp140-Zera^®^-*Delta*TK encoding gp140-Zera^®^ without the tissue plasminogen activator leader (T) and KDEL (K) sequences was generated to investigate if the presence of the leader sequence and KDEL had any impact on localization. The KDEL sequence prevents a protein from being secreted from the ER. Western blotting and immunofluorescence staining of HEK293T cells transfected with these plasmids indicated that there were no obvious differences between gp140-Zera^®^ and gp140-Zera^®^-ΔTK. Thus, the presence of an ER retention signal did not seem to make any notable impact on the localization of gp140-Zera^®^ (data not shown). Protein bodies were observed in HEK293T cells expressing gp140-Zera^®^ but not in those expressing gp140 or Zera^®^-gp140 using transmission electron microscopy (Figure 2C).

As a cost-effective measure for routine production, HEK293 cells stably expressing gp140-Zera^®^ and Zera^®^-gp140 were successfully generated as confirmed by western blot analysis of the cell lysates (Figure 2D and Supplementary Figure S1). Protein bodies were not visible on immunofluorescent staining of stable HEK293 cells expressing Zera^®^-gp140 and gp140-Zera^®^ (results not shown).

### Purification of Zera^®^-Tagged Proteins

**FIGURE 3:**
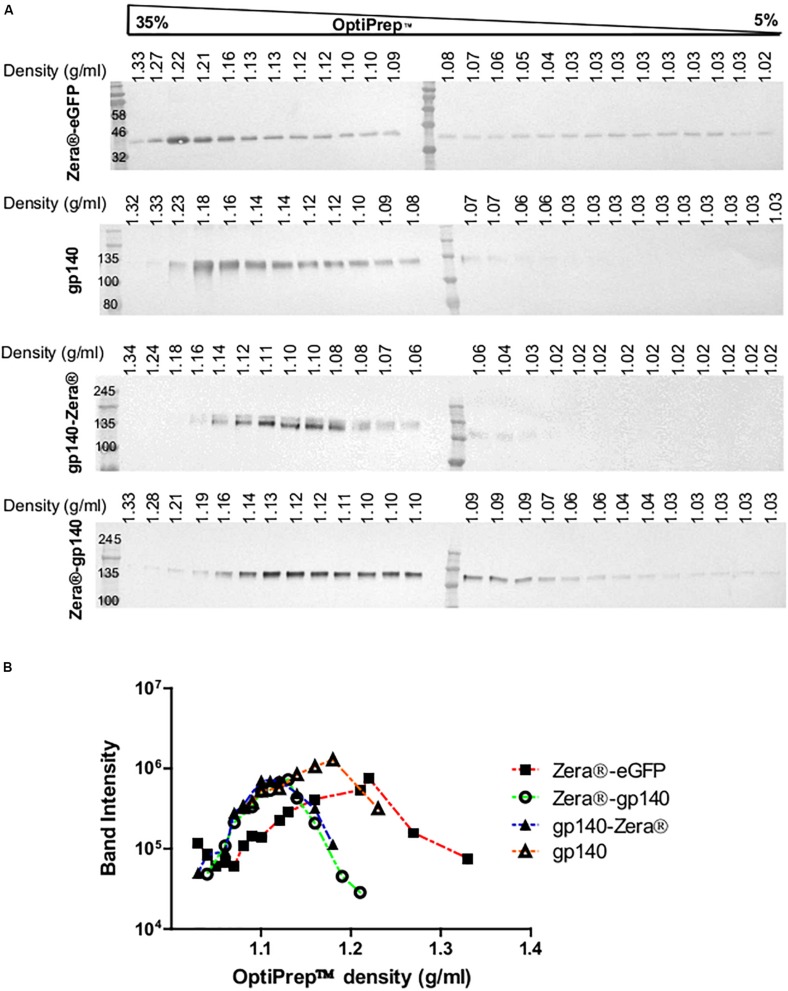
Isopycnic ultracentrifugation of the lysates from HEK293T cells transiently expressing Zera^®^-eGFP and gp140 as well as lysates from HEK293 stable cell lines expressing gp140-Zera^®^ and Zera^®^-gp140. **(A)** Cell lysates were applied on top of a 5–35% continuous OptiPrep^TM^ gradient followed by isopycnic ultracentrifugation. Fractions were analyzed on western blots probed with antibodies to Env or GFP. Refractive indices of each fraction were converted to density (g/ml). **(B)** Band intensities from western blots were plotted against the OptiPrep^TM^ densities.

Previous studies indicated that Zera^®^-tagged proteins accumulate in electron-dense PBs, thus allowing for simple protein recovery using isopycnic ultracentrifugation or low speed rate-zonal centrifugation (Mainieri et al., 2004; Torrent et al., 2009a, b). We evaluated if Zera^®^-induced PBs in cells expressing Zera^®^-gp140, gp140-Zera^®^, or Zera^®^-eGFP could be isolated by isopycnic gradient ultracentrifugation. Clarified lysates from cells expressing Zera^®^-gp140, gp140-Zera^®^, Zera^®^-eGFP and gp140 were fractionated on 5–35% OptiPrep^TM^ gradients, and the resultant fractions were analyzed by western blots probed with anti-GFP and anti-Env (Figure 3A and Supplementary Figure S2). To analyze the distribution of Zera^®^-induced PBs in an OptiPrep^TM^ gradient, band intensities extrapolated from western blots were plotted against OptiPrep^TM^ densities corresponding to each fraction (Figure 3B). As expected, Zera^®^-eGFP was distributed predominantly in fractions corresponding to high OptiPrep^TM^ density (1.21–1.22 g/ml). Both gp140-Zera^®^ and Zera^®^-gp140 were predominantly distributed in a similar range of OptiPrep^TM^ densities (1.11–1.13 g/ml) which was lower than that of Zera^®^-eGFP. Surprisingly, gp140 was predominantly distributed in fractions with relatively higher OptiPrep^TM^ densities (1.14–1.18 g/ml) than those observed for sedimentation of Zera^®^-gp140 and gp140-Zera^®^.

### Antibody Responses to Zera^®^-gp140 and gp140-Zera^®^ in Rabbits

**FIGURE 4:**
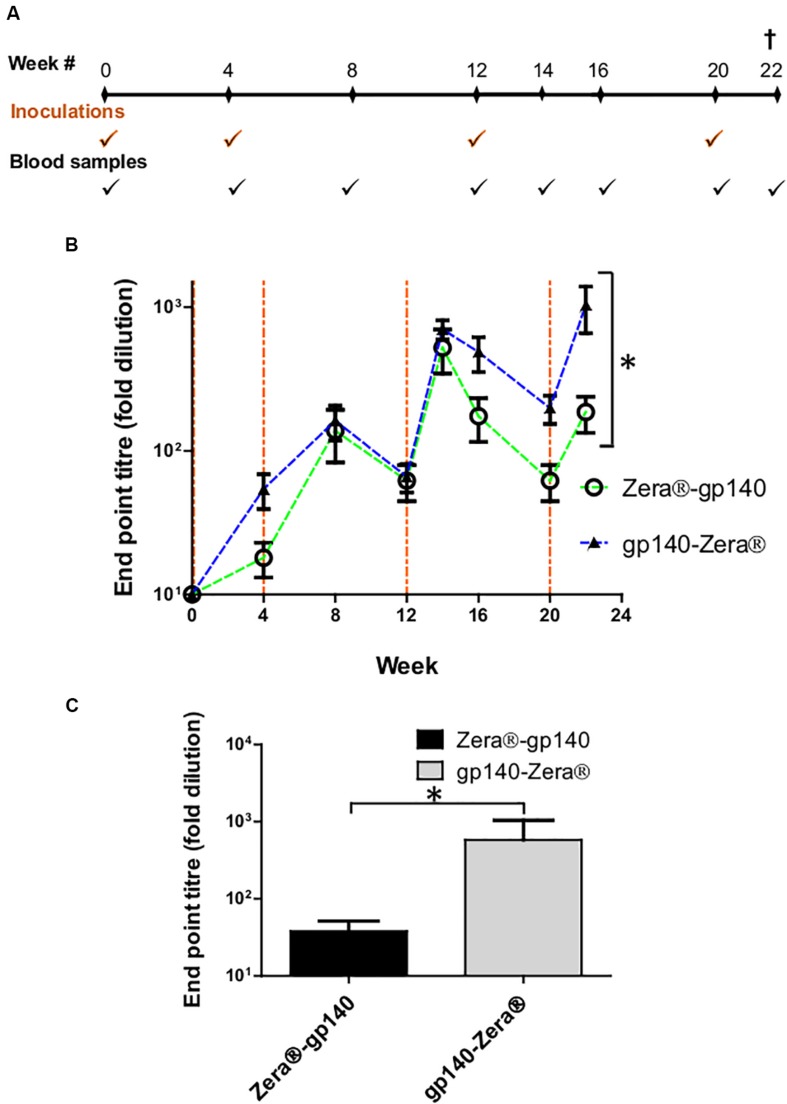
Vaccination schedule and serum characterization. **(A)** Schematic showing the timing of inoculations of two groups of rabbits with Zera^®^-gp140 or gp140-Zera^®^ and collection of blood samples (tick marks). **(B)** Anti-Env antibody titers in sera were quantified in an indirect ELISA where the SEC-purified gp140 trimers were used as the capture antigen. The time points for the inoculation of rabbits with proteins are indicated by vertical dotted lines (orange). Error bars indicate the standard deviation within the group at each time point. The *P*-value (*P = 0.0013) between the two groups was calculated by Two-Way ANOVA, Bonferroni post-test. **(C)** Anti-CAP256 V1V2 scaffold endpoint binding titers in sera (week 22) from rabbits inoculated with gp140-Zera^®^ and Zera^®^-gp140. The *P*-value (*P = 0.2747) was extrapolated from the unpaired *t*-test analysis.

The immunogenicity of gp140-Zera^®^ and Zera^®^-gp140 was evaluated by inoculating two groups of 5 rabbits with approximately 40μg Zera^®^-tagged gp140 protein at weeks 0, 4, 12, and 20 (Figure 4A). Both these protein formulations were administered without any commercial adjuvants.

HIV-1 gp140 binding antibody titers from serum sampled at weeks 4, 8, 12, 14, 16, 20, and 22 were measured in an indirect binding ELISA where SEC-purified gp140 protein was used as the capture antigen (van Diepen et al., 2019). The accumulation of antibodies against gp140 in sera from rabbits inoculated with Zera^®^-gp140 and gp140-Zera^®^ followed a similar trend, where anti-gp140 antibodies were boosted 2 weeks after each protein boost (weeks 14 and 22) (Figure 4B). The anti-gp140 antibody titers in sera from rabbits inoculated with gp140-Zera^®^ reached maximum titers after four protein inoculations at week 22, while rabbits inoculated with Zera^®^-gp140 reached maximum titers after the 3rd protein inoculation at week 14. Notably, at weeks 16–22 anti-gp140 titers in rabbits inoculated with gp140-Zera^®^ were 3 to 6-fold higher than titers observed in rabbits inoculated with Zera^®^-gp140. Interestingly, the overall anti-gp140 endpoint antibody binding titers elicited in rabbits inoculated with gp140-Zera^®^ were significantly higher than those elicited in rabbits inoculated with Zera^®^-gp140 protein (^∗^*P* = 0.0013, Two-Way ANOVA, Bonferroni post-tests).

Binding antibodies to the autologous scaffolded CAP256 SU V1V2 loop were measured using rabbit sera collected at week 22 (2 weeks after the final protein boosts) (Figure 4C). The anti-V1V2 tires in sera from rabbits inoculated with gp140-Zera^®^ were higher than those observed in sera from rabbits inoculated with Zera^®^-gp140; however, the difference was not significant (*P* = 0.2747, unpaired *t*-test analysis).

### Neutralization Assays With Env-Pseudotyped Viruses

**FIGURE 5:**
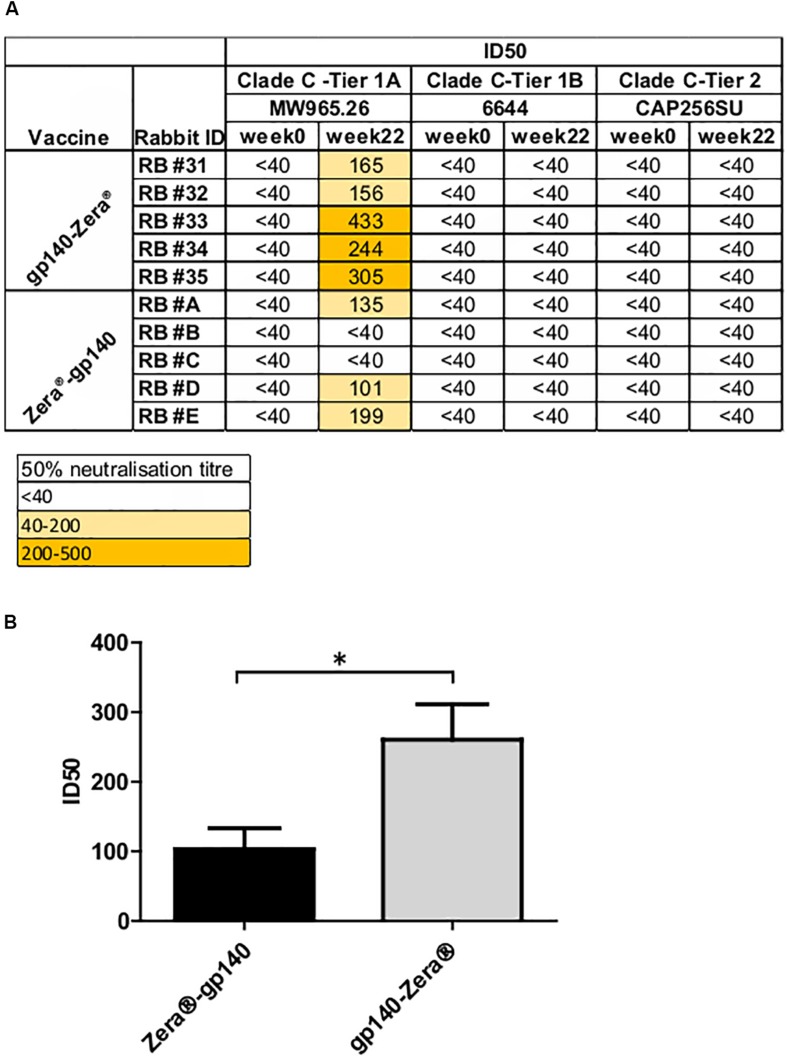
Neutralizing antibody titers elicited in rabbits vaccinated with gp140-Zera^®^ and Zera^®^-gp140. **(A)** The neutralizing activity of sera collected before (week 0) and after inoculation with four doses of gp140-Zera^®^ or Zera^®^-gp140 (week 22) was assayed against a small panel of Clade C pseudoviruses. Virus neutralization was represented as the reciprocal of sera dilutions required to achieve 50% reduction of viral entry into TZM-bl cells (ID50). **(B)** Tier 1 A MW965.26 neutralization titers in sera from rabbits inoculated with gp140-Zera^®^ and Zera^®^-gp140. The *P*-value (*P = 0.0288) was extrapolated from the unpaired *t*-test analysis.

A TZM-bl assay was used to investigate the neutralizing activity of selected sera (weeks 0 and 22) against a small panel of Env-pseudotyped clade C viruses (Figure 5A). All the rabbits inoculated with gp140-Zera^®^ developed neutralizing antibodies against the Malawian Tier 1A pseudovirus (MW965.2) with ID50s ranging between 1:156 and 1:433, compared to only 3/5 of the rabbits vaccinated with Zera^®^-gp140 (ID50 1:101 to 1:135). Rabbits inoculated with gp140-Zera^®^ developed significantly higher neutralizing activity against Tier 1A MW9652.2 virus than rabbits inoculated with Zera^®^-gp140 (Figure 5B). None of the sera from either group neutralized the Tier 1B 6644 or the autologous Tier 2 CAP256SU Env-pseudotyped viruses (Figure 5A).

## Discussion

We previously compared the development of binding and neutralizing antibodies in rabbits inoculated with the soluble HIV-1 CAP256 gp140 formulated in AlhydroGel^®^ and AddaVax^®^ adjuvants (van Diepen et al., 2018). This follow-up study was designed to evaluate whether self-assembling protein nanoparticles (protein bodies) encapsulating CAP256 gp140 could improve the immunogenicity of the glycoprotein. We evaluated the production of protein bodies (PBs) by fusing the sequence encoding Zera^®^, a proline-rich domain derived from the γ-zein storage protein, to either the C- or N-terminus of HIV-1 CAP256 gp140. To our knowledge, this is the first time the possibility of formation of protein bodies encapsulating HIV Env has been explored, and this is the largest glycoprotein (±140 kDa) to be fused to Zera^®^ as it is larger than the ectodomain of influenza A virus subtype 5 HA (±75 kDa) previously fused by others (Hofbauer et al., 2016).

The ability of gp140-Zera^®^ and Zera^®^-gp140 to form PBs was compared to Zera^®^-eGFP, which has previously been shown to form PBs in mammalian cells (Torrent et al., 2009a). Immunofluorescent staining and confocal microscopy of HeLa cells expressing gp140-Zera^®^ and Zera^®^-gp140 indicated that gp140-Zera^®^ formed comparatively smaller and fewer spherical PB-like structures than Zera^®^-eGFP, which was solely expressed as distinct PBs. In cells expressing Zera^®^-gp140, Env appeared diffused in cells without any observable PB-like structures. Previous studies indicated that zein-derived sequences fused to either the C- or N-terminus of the protein of interest induced the formation of PBs in a wide spectrum of eukaryotic expression systems (de Virgilio et al., 2008; Torrent et al., 2009a; Joseph et al., 2012; Whitehead et al., 2014; Hofbauer et al., 2016). However, not all proteins fused to zein-derived sequences are able to assemble into PBs. For example, the HIV negative factor (Nef) protein fused to the γ-zein domain (zein-Nef) did not form PBs in plants since it was rapidly degraded in the ER. On the other hand, PBs stably encapsulating Nef were formed when Nef was fused to zeolin, a chimeric domain composed of the γ-zein (same sequence used for zein-Nef) as well as the bean vascuolar storage protein phaseolin (Mainieri et al., 2004; de Virgilio et al., 2008). The authors reasoned that unlike the correctly folded zeolin-Nef, zein-Nef may have been recognized by the ER quality control machinery as a structurally defective protein and was thus directed to the degradation pathway in the ER before being able to assemble into PBs. This suggested that regardless of the presence of zein-derived sequences, if the fusion protein is misfolded zein sequences do not afford escape from endoplasmic reticulum-associated degradation (ERAD). Therefore, misfolded zein-Nef did not accumulate to levels sufficient for assembly into otherwise physiologically inert PBs. The misfolding of zein-Nef was thought to be due to the formation of aberrant disulfide bonds between 3 Cys residues in the Nef sequence with 6 Cys residues of zein sequence in the ER (de Virgilio et al., 2008). A number of different reports have shown that HIV-1 Env proteins can contain aberrant disulfide bonds which lead to the misfolding and aggregation of the protein (Kassa et al., 2013; Ringe et al., 2013, 2015; Go et al., 2015).

We showed that although higher levels of Zera^®^-gp140 were produced than gp140-Zera^®^, Zera^®^-gp140 failed to assemble into PBs. The folding of Zera^®^-tagged gp140 proteins was not assessed; however, the failure to form Zera^®^-gp140 PBs might also be due to the formation of aberrant disulfide bridges between Zera^®^ and gp140 Cys residues. Site-directed mutagenesis of individual cysteine residues of Zera^®^ indicated that in addition to the amphipathic (PPPVHL)_8_ repeat of Zera^®^, the six cysteine residues flanking this repeat are required for disulfide cross-linking of Zera^®^ sequences during oligomerization into PBs (Llop-Tous et al., 2010). There are 18 cysteine residues in HIV-1 CAP256 gp140 which in the ER, could potentially form aberrant disulfide bonds as a result of isomerization with the cysteine residues of Zera^®^, thus forming misfolded protein and inhibiting the multimerization into PBs or trimers. Such misfolding is more likely for Zera^®^-gp140 where no flexible linker was included between Zera^®^ and gp140 sequences to allow for independent but cooperative folding. In comparison to well folded native-like trimers, non-native gp140 trimers were reported to contain a substantial proportion of aberrant disulfide bonds (Go et al., 2015). Thus, we reasoned that if aberrant disulfide bond formation is a common occurrence for recombinant gp140 proteins, fusion to the Cys-rich Zera^®^ coding sequence would probably exacerbate this phenomenon.

Ceresoli et al. (2016) reported another case where zein-tagged recombinant human bone morphogenetic protein 2 active dimers (zein-rhBMP2ad) accumulated at higher levels than the native soluble protein (hBMP2nat) but failed to induce PBs. The authors did not think the failure to form PBs was due to misfolding that could be caused by the possible aberrant disulfide bridges (hBMP2ad contains 7 Cys residues). Instead, they reasoned that the failure of zein-rhBMP2ad to form PBs may have been due to *N*-glycosylation that increased the solubility of rhBMP2ad and prevented PB formation (Ceresoli et al., 2016). We note that gp140 contains ≈28 *N*-glycosylation sites which could favor solubility of the Zera^®^-tagged gp140, thus preventing optimal assembly into PBs.

Zera^®^ was also fused on the C-terminus of gp140 to form gp140-Zera^®^, because it was reasoned that this would not occlude the gp120 subunits which contain important epitopes for eliciting antibodies. Even though gp140-Zera^®^ appeared to form some small PB-like structures in transiently transfected cells, expression of gp140-Zera^®^ was lower than Zera^®^-gp140. This pattern was also observed by de Virgilio et al. (2008) who showed that expression levels of zein-Nef were higher than Nef-zein, which was barely detectable, indicating that the location of Zera^®^ can affect protein accumulation. Additionally, the complexity of and large size of HIV Env may have hindered the optimal formation of Zera^®^-induced nanoparticles. He et al. (2016) showed that when gp140 was fused to the 60-meric lumazine synthase (LS), nanoparticles were not formed. They reasoned that the large size and spacing of Env antigens could limit its display on self-assembling nanoparticles (He et al., 2016). It is also possible that the protein bodies formed with gp140-Zera^®^ are too labile and are broken up during extraction. To our knowledge the largest Zera^®^-fusion protein that has been shown to form protein bodies is the 70kDa influenza H5 (Hofbauer et al., 2016).

It is also possible that in our case the concentration of gp140 may not have been sufficient to drive the formation of PBs: Saberianfar et al. (2015) have reported that the fusion of GFP to other PB-inducing tags, hydrophobin-i and elastin-like polypeptides, indicated that the formation of protein bodies is a concentration-dependent mechanism where the accumulation of GFP to a minimum of 0.2% of the total soluble protein (TSP) was required for oligomerization into PBs. The size of these PBs simultaneously increased over time with an increase in protein concentration. Interestingly, it was also observed that if GFP accumulated to a value higher than 6.5% of the TSP, PB-like structures were observed regardless of the presence or absence of the PB-inducing fusion tags (Gutierrez et al., 2013; Saberianfar et al., 2015). Unlike other proteins that have been fused to PB-inducing tags, gp140 is a complex protein that is difficult to express and it is possible that it might not have reached the threshold concentration required for optimal assembly into PBs.

Previous studies have shown that zein-derived domains induce the formation of electron dense PBs with a diameter of 0.5–2 μm which settle at a density of approximately 1.18–1.26 g/ml during subcellular fractionation of a density gradient, thus allowing for simple protein recovery using rate zonal or isopycnic density ultracentrifugation (Mainieri et al., 2004; Torrent et al., 2009a). In theory, if Zera^®^-tagged gp140 is encapsulated in PBs, it would be expected to be more dense than the soluble gp140. However, Zera^®^-gp140 and gp140-Zera^®^ were predominantly detected in fractions with densities ranging from 1.1 to 1.13 g/ml. These densities are lower than 1.18–1.26 g/ml reported by Torrent et al. (2009a). Gp140-Zera^®^ and Zera^®^-gp140 fractionated at densities similar to the approximate OptiPrep^TM^ density range for the ER, which ties in well with the observed co-localization of these proteins with calnexin. These results are in accordance with a previous study which showed that, unlike zeolin-Nef that formed PBs, zein-Nef did not efficiently form PBs and could not be distinguished from the ER (de Virgilio et al., 2008). Zein-rhBMP2ad also did not form protein bodies but was retained in the ER (Ceresoli et al., 2016). Our findings present another case where Zera^®^-tagged gp140 did not efficiently form PBs but was associated with the ER, thereby separating and settling in fractions of densities previously noted where ER membrane fragment settles. As the species of Zera^®^-tagged gp140 protein spanned a large range of different sizes/densities, it was not feasible to take advantage of the high-density properties inherent to PBs for large scale protein preparations by isopycnic and rate zonal ultracentrifugation. As a result, Zera^®^-tagged proteins were concentrated by pelleting of cell lysates by ultracentrifugation to provide sufficient amounts for immunogenicity studies.

The immunogenicity of Zera^®^-gp140 and gp140-Zera^®^ was tested in rabbits to evaluate the adjuvant activity of Zera^®^. Both proteins elicited high titers of HIV-1 Env binding antibodies of similar levels after 3 immunizations but rabbits vaccinated with gp140-Zera^®^ had significantly higher titer than those vaccinated with Zera^®^-gp140 after 4 immunizations. Since PBs were not efficiently formed, the adjuvanting properties observed herein may be conferred by Zera^®^, as has been shown when Zera^®^ PBs alone were an efficient adjuvant for an engineered HPV-16 E7 protein (Whitehead et al., 2014). Sera from rabbits inoculated with Zera^®^-gp140 and gp140-Zera^®^ showed equivalent binding titers to the CAP256 V1V2 scaffold antigen. Zera^®^-tagged gp140 elicited V1V2 titers similar to those observed in historical sera from rabbits inoculated with gp140 (van Diepen et al., 2018). This indicated that the presence of Zera^®^ did not occlude gp140 epitopes responsible for triggering V1V2-specific responses. The V1V2 binding antibodies are of particular relevance in the context of vaccine development because they correlated with protection against HIV acquisition in the RV144 trial (Haynes et al., 2012). It would be interesting to evaluate the Fc-mediated effector functions such as antibody dependent cellular cytotoxicity (ADCC) of these antibodies, the secondary correlate of protection in the RV144 trial (Haynes et al., 2012).

One of the goals of HIV vaccine research is to design immunogens that elicit antibodies that potently neutralize a broad range of Tier 2 circulating viruses. In a luciferase reporter gene neutralization assay, sera from rabbits inoculated with g140-Zera^®^ elicited significantly higher Tier 1A neutralizing antibodies than sera from rabbits inoculated with Zera^®^-gp140. However, neither protein elicited Tier 1B or autologous Tier 2 neutralizing antibodies which may be explained by limited amounts of conformationally appropriate gp140. H5-zein (Hofbauer et al., 2016) and the plant-produced monomeric HA fused to ELP (H5-ELP) failed to elicit neutralizing antibodies, but Nabs were elicited when H5-ELP was stabilized into trimers by the addition of the GCN4-pII isoleucine-zipper trimerization motif directly downstream of the H5 ectodomain (Harbury et al., 1993; Phan et al., 2013). Likewise, proper trimerization of HIV Env is mandatory to yield native-like trimers that assume a conformational configuration that optimally present epitopes for induction of Tier 2-neutralizing antibodies (Sanders et al., 2015; de Taeye et al., 2016; Sanders and Moore, 2017). Additionally, the chances of eliciting good neutralization responses could be improved by selectively using trimeric Env separated from aggregates, dimeric and monomeric Env species using size exclusion chromatography. However, during assembly into PBs, there is no way of ensuring that monomeric, dimeric and aggregated Env species are not encapsulated together with trimeric Env.

The inability to control the quality of Env or of nanoparticles can be a drawback of the use of *in vivo*-assembling protein nanoparticles. However, this can be overcome by using two-component platforms where native-like, trimeric Env and nanoparticles are expressed and purified separately and then assembled *in vitro* (Brinkkemper and Sliepen, 2019).

## Conclusion

This was an exploratory study to assess whether we could take advantage of the beneficial properties of Zera^®^ protein bodies for the HIV-1 envelope glycoprotein. A considerable number of assays were done to evaluate the formation of HIV Env PBs. However, our results indicated that Zera^®^ did not efficiently form PBs when fused to the HIV-1 gp140 protein. This study has shown that HIV-1 envelope proteins, tagged with Zera^®^ at the N and C termini, elicited high titers of gp140 and V1V2 binding antibodies in rabbits. In addition, both gp140-Zera^®^ and Zera^®^-gp140 elicited low levels of Tier 1 neutralizing antibodies. Further studies need to be carried out to determine whether these antibodies have Fc-mediated effector functions.

## Data Availability Statement

All datasets generated for this study are included in the article/Supplementary Material.

## Ethics Statement

The animal study was reviewed and approved by University of Cape Town Animal Ethics Committee.

## Author Contributions

RC and PX designed the project. PX performed all experiments, data analysis, and drafted the manuscript. RC, AM, A-LW, and ER supervised the study and participated in drafting the manuscript. ER initiated the study. EM and MD assisted with some experiments and data analysis, and editing of the manuscript. All authors read and approved the final manuscript.

## Conflict of Interest

The authors declare that the research was conducted in the absence of any commercial or financial relationships that could be construed as a potential conflict of interest.
