# Supplementary figures and images for: Characterization and Immunogenicity of HIV Envelope gp140 Zera® Tagged Antigens
Source: Front Bioeng Biotechnol. 2020 Apr 9;8:321. doi: 10.3389/fbioe.2020.00321 (PMC7160593; doi:10.3389/fbioe.2020.00321)

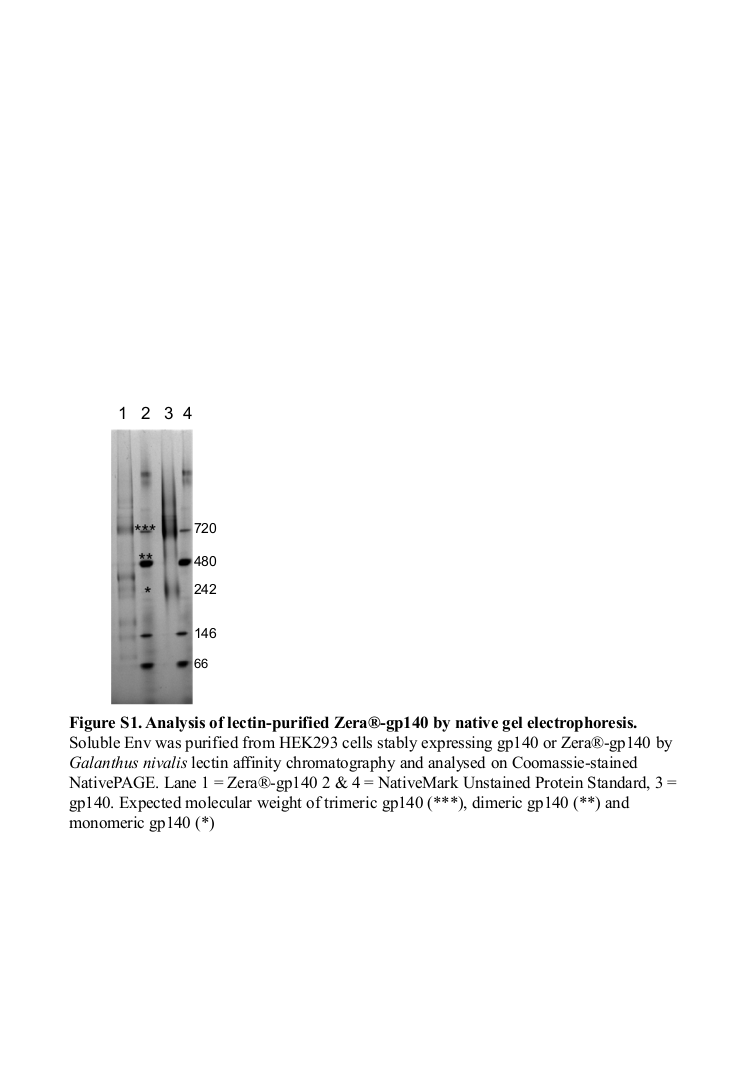

Supplement: Supplementary file 1 [file Image_1.tiff]

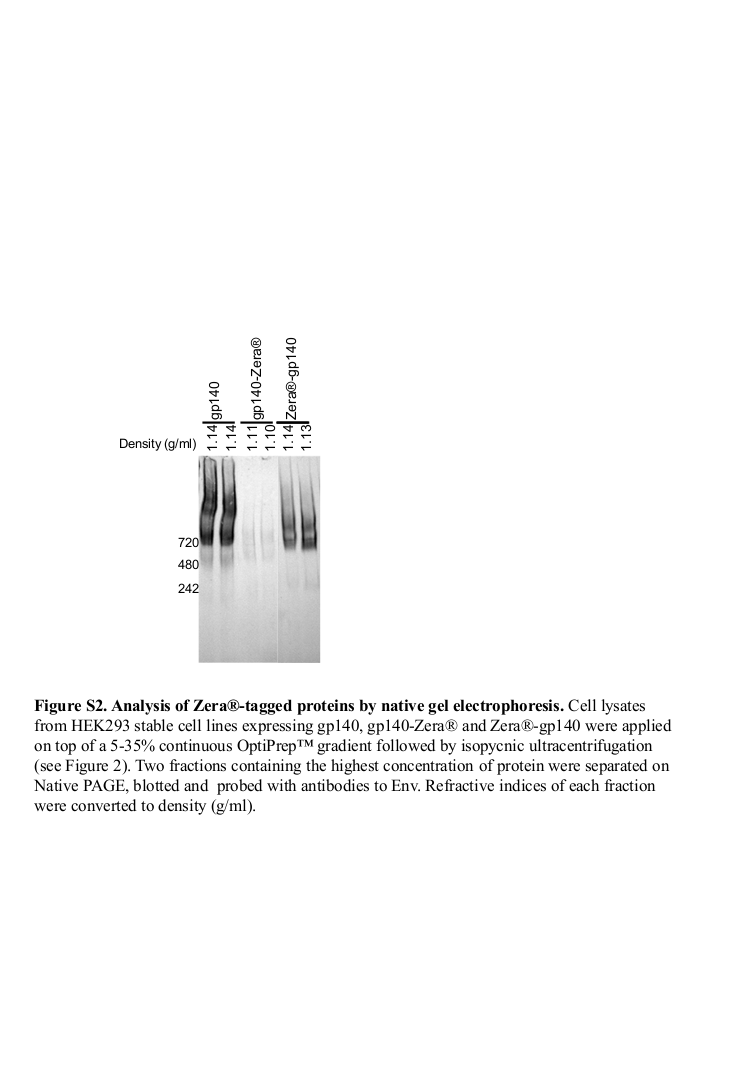

Supplement: Supplementary file 2 [file Image_2.tiff]
